# Genetic structure and bio-climatic modeling support allopatric over parapatric speciation along a latitudinal gradient

**DOI:** 10.1186/1471-2148-12-149

**Published:** 2012-08-20

**Authors:** Maurizio Rossetto, Chris B Allen, Katie AG Thurlby, Peter H Weston, Melita L Milner

**Affiliations:** 1 National Herbarium of NSW, Mrs Macquaries Road, Sydney, NSW, 2000, Australia; 2 Evolution, Ecology and Genetics, Research School of Biology, The Australian National University, Canberra, ACT, 0200, Australia

**Keywords:** Allopatry, Environmental niche models, Parapatry, Secondary hybridisation, Speciation, Proteaceae, *Telopea*, waratah

## Abstract

**Background:**

Four of the five species of *Telopea* (Proteaceae) are distributed in a latitudinal replacement pattern on the south-eastern Australian mainland. In similar circumstances, a simple allopatric speciation model that identifies the origins of genetic isolation within temporal geographic separation is considered as the default model. However, secondary contact between differentiated lineages can result in similar distributional patterns to those arising from a process of parapatric speciation (where gene flow between lineages remains uninterrupted during differentiation). Our aim was to use the characteristic distributional patterns in *Telopea* to test whether it reflected the evolutionary models of allopatric or parapatric speciation. Using a combination of genetic evidence and environmental niche modelling, we focused on three main questions: do currently described geographic borders coincide with genetic and environmental boundaries; are there hybrid zones in areas of secondary contact between closely related species; did species distributions contract during the last glacial maximum resulting in distributional gaps even where overlap and hybridisation currently occur?

**Results:**

Total genomic DNA was extracted from 619 individuals sampled from 36 populations representing the four species. Seven nuclear microsatellites (nSSR) and six chloroplast microsatellites (cpSSR) were amplified across all populations. Genetic structure and the signature of admixture in overlap zones was described using the Bayesian clustering methods implemented in STUCTURE and NewHybrids respectively. Relationships between chlorotypes were reconstructed as a median-joining network. Environmental niche models were produced for all species using environmental parameters from both the present day and the last glacial maximum (LGM).

The nSSR loci amplified a total of 154 alleles, while data for the cpSSR loci produced a network of six chlorotypes. STRUCTURE revealed an optimum number of five clusters corresponding to the four recognised species with the additional division of *T. speciosissima* into populations north and south of the Shoalhaven River valley. Unexpectedly, the northern disjunct population of *T. oreades* grouped with *T. mongaensis* and was identified as a hybrid swarm by the Bayesian assignment test implemented in NewHybrids. Present day and LGM environmental niche models differed dramatically, suggesting that distributions of all species had repeatedly expanded and contracted in response to Pleistocene climatic oscillations and confirming strongly marked historical distributional gaps among taxes.

**Conclusions:**

Genetic structure and bio-climatic modeling results are more consistent with a history of allopatric speciation followed by repeated episodes of secondary contact and localised hybridisation, rather than with parapatric speciation. This study on *Telopea* shows that the evidence for temporal exclusion of gene flow can be found even outside obvious geographical contexts, and that it is possible to make significant progress towards excluding parapatric speciation as a contributing evolutionary process.

## Background

Models of speciation are generally categorised on the role and nature of geographic isolation during the origination of reproductive barriers between differentiating populations. Usually, allopatric speciation is considered as the default model since it specifies a simpler, more biologically plausible process than other models [1,2]. In an allopatric model of speciation, genetic isolation resulting from temporal geographic separation can lead to the evolution of reproductive barriers and speciation. Consequently, biogeographic patterns are of particular interest when attempting to test allopatric models [3]. In contrast, the parapatric and sympatric speciation models involve the differentiation of lineages despite the possibility for ongoing exchange of genetic material. In these models, selective barriers must have a strong impact in order to preserve between-lineage divergence [4,5].

The spatial arrangement of genetically divergent populations is important in allopatric speciation, as it involves the geographic separation of a previously continuous metapopulation by geological or climatic causes, or a founder event resulting from long distance dispersal [6]. Lack of gene flow, drift and secondary adaptation to local environmental conditions among geographically separated populations can eventually result in morphologically and genetically distinct lineages. However, current isolating mechanisms are not necessarily the same as those that initiated speciation and, as a result, secondary contact and hybridisation can ensue [3]. Although the presence of hybrid fronts between closely related lineages can confirm allopatric speciation with secondary contact, such fronts are difficult to differentiate from zones of primary parapatric speciation. In fact, any mechanism that can cause divergence in allopatry can theoretically also act in parapatry, as long as the selective gradient acting on differentiation is sufficiently strong to counterbalance continuing gene flow [1].

Here, we aim to identify the main speciation mechanism involved in the diversification of the plant genus *Telopea* (the waratahs; Proteaceae). *Telopea* is a genus comprising five species of long-lived, lignotuberous, sclerophyllous, bird-pollinated, wind-dispersed shrubs and small trees distributed in mesic environments of south eastern Australia, including Tasmania. They live in eucalypt-dominated forests and woodlands, growing on acidic, nutrient-poor, well drained soils derived from sandstone, granite, chert or quartzite from sea level in the Sydney region to 1200 m altitude in subalpine habitats in Tasmania. Weston & Crisp [7] reconstructed *Telopea* phylogeny in a cladistic analysis of morphological characters and the species limits of the four mainland Australian species were tested morphometrically by Crisp & Weston [8].

**Figure 1:**
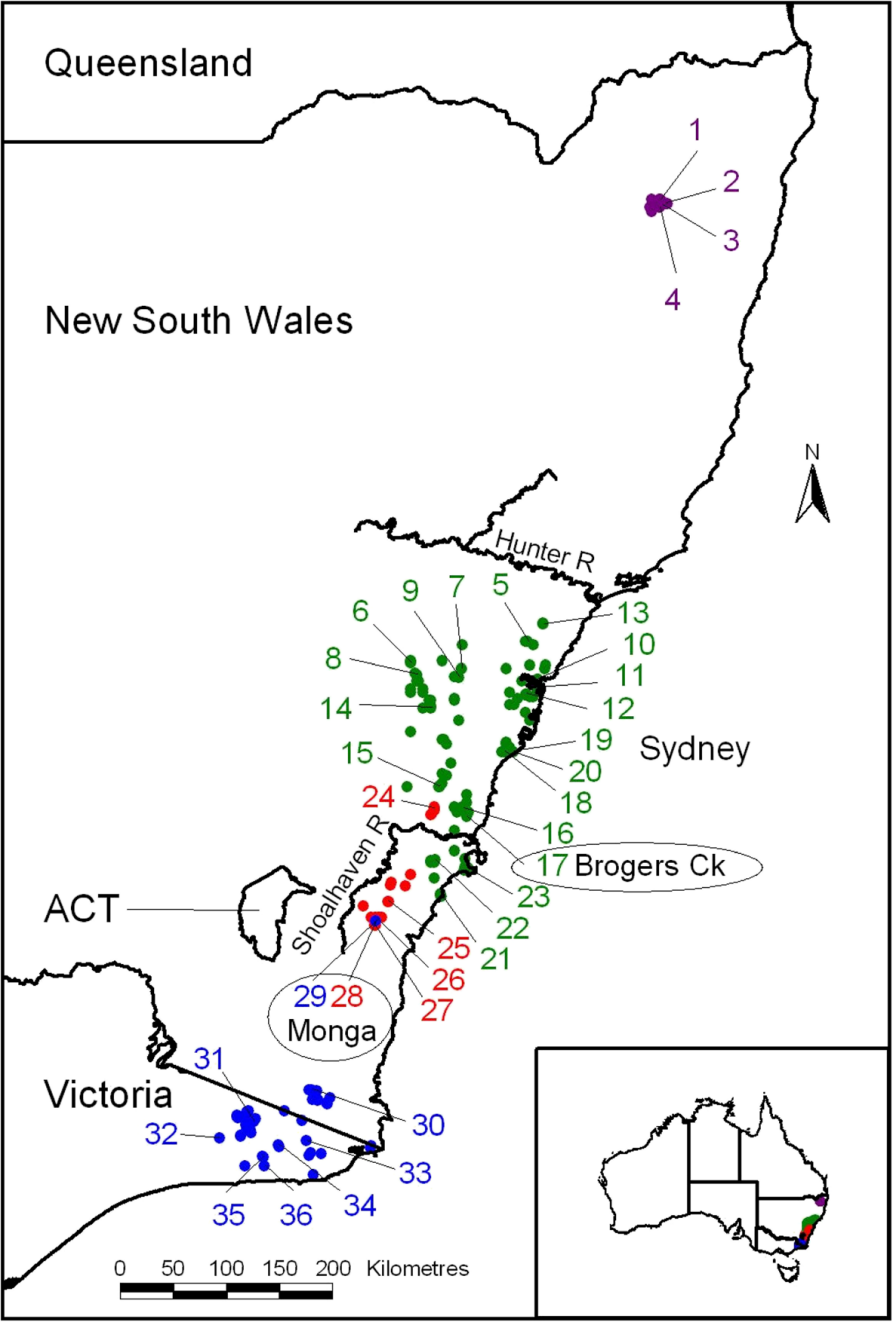
**Distribution map for the four *Telopea *species investigated in this study.** Map showing all verified distributional records (used as training data for the Maxent models) for the four study species (purple *T. aspera*; green *T. speciosissima*; red *T. mongaensis*; blue *T. oreades*). The major geographic points of interest discussed in the text are presented.

Phylogenetically, the four recognised mainland taxa are grouped into two morphologically distinct clades comprising *T. speciosissima* and *T. aspera* to the north, and *T. oreades* and *T. mongaensis* to the south. Their distribution follows a latitudinal pattern ranging from southern Victoria to northern New South Wales (Figure 1). The species are mostly separated by large distributional gaps. The exceptions are a site at Monga National Park (NP), where a geographically disjunct population of *T. oreades* is found in sympatry with *T. mongaensis*, and an area also south of the Sydney Basin where a potential distributional overlap between the latter species and *T. speciosissima* occurs (recent fieldwork has located a single morphologically intermediate individual).

Although terminally-winged, the seeds of *Telopea* are not easily dispersed and tend to germinate in proximity to the maternal parent [9]. As a result, the latitudinal distribution of the genus suggests that interruptions of connectivity between once continuously distributed lineages could have contributed to allopatric speciation with the development of morphological and adaptive differentiation likely to have been secondary events. In a previous landscape genetic study on *T. speciosissima*, we suggested that substrate-mediated allopatry and temperature-dependent reproductive barriers were likely to affect temporal changes in connectivity among populations [10]. While genetic differentiation in the south corresponded to a distributional gap caused by changes in edaphic conditions, altitudinal constraints in gene flow were the consequence of a strong association between temperature and flowering time.

In south-eastern Australia, the glacial cycles of the Quaternary shaped the distribution of plants and plant communities by increasing aridity, fire frequency and fire intensity [11]. The limited fossil record suggests that dynamic successional processes affected local vegetation types, and that localised moist refugia along the Great Dividing Range provided some protection against localised extinctions [12,13]. Plant distributional changes during the Quaternary have been particularly well documented in northern latitudes [14,15] and more recently in eastern Australia [16]. Similar temporal changes can also be expected for *Telopea*. The extent to which a species’ distribution can respond to changing climatic conditions is affected by niche breadth, whilst the ability of a species to establish in new climatically suitable areas is dependant upon numerous ecological factors, including dispersal ability and competitive advantage. The use of environmental niche modeling (ENM) to investigate historical environmental suitability in combination with molecular data can enhance our understanding of temporal changes in population dynamics [17]. Here we integrate genetic and bio-climatic methods to investigate speciation across all mainland species of *Telopea* and test the alternative scenarios of parapatric or allopatric speciation. In particular we focus on three main questions:

1. Do currently described geographic borders coincide with genetic and environmental boundaries? Distributional and morphological data mostly support this [8]. Here we aim to validate between-species distinctiveness by combining present-day genetic (nSSRs, cpSSRs) and bio-climatic (ENM) analyses.

2. Are there hybrid zones in areas of secondary contact between closely related species? Here we use targeted population genetic data in overlap zones to investigate hybridisation between species. A previous study on *T. speciosissima* suggested that admixture between previously differentiated populations can take place as a consequence of the post-LGM re-establishment of gene flow caused by temperature-mediated phenological shifts along the altitudinal gradients [10].

3. Did species distribution contract during the last glacial maximum, resulting in distributional gaps even where overlap and hybridisation currently occur? We use ENM to predict historical ranges for the four species and gather evidence for distributional discontinuities. The existence of such patterns would provide support for secondary rather than primary contact zones and consequently favour a scenario of allopatric over parapatric speciation [3].

## Methods

### Study species

Five species of *Telopea* are distributed from the Gibraltar Range in south-eastern mainland Australia (29° 28’ S) to southern Tasmania (43° 33’ S). Of the four mainland species, *T. aspera* in the north is the only one completely separated from its nearest congeners by about 400 km, while the other three have potential contact points at the extremes of their distributions (Figure 1). All species have naturally fragmented distributions, of which the most intriguing is that of *T. oreades*, the most northern population of which overlaps with the most southerly population of *T. mongaensis* but is located over 160 km from the next most northerly population of *T. oreades*[8]. *Telopea mongaensis* also shows a significant internal gap of over 45 km imposed by the Shoalhaven River valley, with its most north-easterly populations occurring within 20 km of the nearest populations of *T. speciosissima*. *Telopea speciosissima* is the most widespread species with populations south of the Shoalhaven River valley being isolated from those on the northern side by a over 30 km [10].

According to the morphology-based cladistic analysis of Proteaceae subtribe Embothriinae published by Weston & Crisp [7], a clade of *T. speciosissima* and *T. aspera* is sister to a clade including *T. truncata* (Tasmania), *T. oreades* and *T. mongaensis*. The *speciosissima-aspera* clade is characterised by three macromorphological synapomorphies: presence of marginal leaf teeth (versus entire or lobed margins), and enlarged, bright red involucral bracts (versus small and green, to dusky pink involucral bracts). The *truncata-oreades-mongaensis* clade is characterised by basitonic inflorescences (flowers open from the tip of the inflorescence towards the base, in contrast to the acrotonic inflorescences of the *speciosissima-aspera* clade and outgroups, in which flowers open from the base towards the tip). *Telopea oreades* and *T. mongaensis* form a clade characterised by markedly bicolorous perianths in which the adaxial surfaces are a much brighter shade of red than the abaxial surfaces, in contrast to the concolorous perianths in other *Telopea* species.

### Sampling, DNA extractions and PCRs

Sampling was aimed at obtaining an account of the genetic diversity across the entire distribution of mainland species (Table 1). Leaf material was collected from a total of 619 individuals, and total genomic DNA was extracted using DNeasy® 96 plant kits (QIAGEN®, Hilden, Germany).

**Table 1:** Study sites

	**Species**	**Population**	**N**	**Latitude**	**Longitude**
1	*T. aspera*	Washpool NP	20	29°29.11'S	152°19.39'E
2	*T. aspera*	Gibraltar Range	22	29°30.8'S	152°21.75'E
3	*T. aspera*	Mulligans Rd	20	29°31.73'S	152°20.19'E
4	*T. aspera*	Anvil Rock	22	29°33.1'S	152°18.88'E
5	*T. speciosissima*	Kulnura	21	33°12.92'S	151°11.92'E
6	*T. speciosissima*	Newnes Forest	20	33°23.67'S	150°12.78'E
7	*T. speciosissima*	Mountain Lagoon	20	33°26.82'S	150°38.55'E
8	*T. speciosissima*	Bell Line Rd	20	33°29.93'S	150°16.08'E
9	*T. speciosissima*	Kurrajong Heights	10	33°31.13'S	150°37.22'E
10	*T. speciosissima*	Patonga	19	33°32.23'S	151°17.05'E
11	*T. speciosissima*	West Head	15	33°36.57'S	151°16.52'E
12	*T. speciosissima*	Duffys Forest	22	33°39.68'S	151°11.5'E
13	*T. speciosissima*	Watagan SF	21	33°4.37'S	151°20.05'E
14	*T. speciosissima*	Kings Tableland	21	33°45.97'S	150°22.93'E
15	*T. speciosissima*	Mt. Alexandra	20	34°26.58'S	150°27.23'E
16	*T. speciosissima*	Carrington Falls	19	34°37.53'S	150°39.32'E
17	*T. speciosissima*	Brogers Creek	8	34°42.00'S	150°40.60'E
18	*T. speciosissima*	Waterfall Flat	15	34°8.83'S	151°0.33'E
19	*T. speciosissima*	Bottle Forest	11	34°8.92'S	151°4.62'E
20	*T. speciosissima*	Curra Moors	10	34°8.92'S	151°4.62'E
21	*T. speciosissima*	Ulladulla	21	35°22.15'S	150°28.4'E
22	*T. speciosissima*	Turpentine Range	21	35°3.72'S	150°25.42'E
23	*T. speciosissima*	Jervis Bay	20	35°7.93'S	150°41.1'E
24	*T. mongaensis*	Gunrock Creek	10	34°37.75'S	150°24.72'E
25	*T. mongaensis*	Budawangs	20	35°24.74'S	150°1.16'E
26	*T. mongaensis*	Dasyurus PA	20	35°33.65'S	149°55.32'E
27	*T. mongaensis*	River Forest Rd	11	35°37.05'S	149°54.7'E
28	*T. mongaensis*	Monga NP	10	35°37.93'S	149°54.73'E
29	*T. oreades*	Monga NP	29	35°37.93'S	149°54.73'E
30	*T. oreades*	Waratah Creek	20	37°0.31'S	149°22.75'E
31	*T. oreades*	Errinundra NP	20	37°16.51'S	148°53.23'E
32	*T. oreades*	Bonang Rd	19	37°24.98'S	148°36.18'E
33	*T. oreades*	Cooaggalah Rd	10	37°26.06'S	149°19.99'E
34	*T. oreades*	Combeinbar Trail	20	37°28.29'S	149°5.91'E
35	*T. oreades*	Lind NP	5	37°34.39'S	148°58.02'E
36	*T. oreades*	Mt Bemm	7	37°38.82'S	148°58.71'E

Name, sample size (N) and location of each *Telopea* population included in this study.

Seven nuclear microsatellites (nSSR) specifically developed for *T. speciosissima* (TS03bgt, TS04bgt, TS12bgt, TS13bgt, TS18bgt, TS23bgt and TS27bgt) were amplified across all individuals using the PCR protocols of Porter *et al*. [18]. Genotyping results were checked with Microchecker [19] for evidence of scoring errors due to stuttering, large allele dropout and null alleles with ranked-based and binomial-based estimates showing no evidence of scoring error.

Chloroplast microsatellites (cpSSR) were amplified from three individuals per population, using *ccmp10*[20], *psb*A [21], *trn*G (UCC)ex1 – *atp*A [22], *Lomcp1Lomcp2* and *Lomcp3* (M Milner *pers. com.*). Reaction conditions were as described in Ebert & Peakall [22] using Schuelke’s [23] method of fluorescently-labelled M13 forward primers. Amplification products were visualized using a 3130xl Genetic Analyzer (Applied Biosystems) and scored for size using GeneMapper 3.7 (Applied Biosystems).

To test genotyping accuracy approximately 20% of all PCR reactions were repeated for each primer pair across random individuals. Less than 5% of those PCR repeats identified errors that needed confirmation through replication of PCR and genotyping. Finally, to verify that the amplified loci indeed contained the expected nSSR repeats across all *Telopea* species and to verify the accuracy of allelic sizes, representatives of species/locus combinations were sequenced [GenBank accession no. JF931649-JF931672]. Unique cpSSR allele sizes were also sequenced across species to ascertain that the variation was due to mononucleotide repeat regions [GenBank accession no. JQ778988-JQ779019].

### Defining genetic boundaries

Species-level averages of nSSR allelic diversity (A), expected (H_e_) and observed (H_o_) heterozygosities as well as number of private alleles (A_p_) were calculated using GDA 1.1 [24]. In order to avoid bias caused by uneven sampling [25], a standardized estimate of allelic richness (R_63_) independent of sample size [26] was calculated using the program FSTAT 2.9.3 [27]. Pariwise F_st_[28] values were also obtained through FSTAT and used to provide within and between species averages.

We used the Bayesian clustering method described by Pritchard *et al*. [29] and implemented in STUCTURE 2.3.3 on the nSSR data to identify genetically differentiated groups of individuals in the absence of preliminary information on group boundaries. The model assumes the existence of *K* clusters (the real number being unknown) and uses the allelic frequencies at each locus to assign individuals to these clusters through a Markov chain Monte Carlo (MCMC) probabilistic approach in which individuals are assigned to clusters so as to maximize Hardy-Weinberg equilibrium within populations. All analyses were based on 10 independent runs for each *K* value, with each individual run being based on 2.5x10^5^ MCMC iterations following a burn-in period of 5x10^4^ iterations without prior information on the taxonomy or the locality of origin of the individuals sampled. The admixture frequency model was run under the assumption of correlated allele frequencies to improve clustering of related lineages and identify possible hybridisation patterns [30]. The optimal number of clusters was verified using the Δ*K* statistical approach suggested by Evanno *et al*. [31].

After a preliminary test aimed at finding a suitable range for *K* and the optimal burn-in period, we tested *K* from 1 to 20 for the full dataset including the four species under study. We also tested *K* from 1 to 12 for the dataset including only *T. oreades* and *T. mongaensis* to specifically investigate assignment patterns for the only sympatric individuals located at the Monga NP site.

Analysis of molecular variance (AMOVA; [32]) was then used to quantify variance components and the significance of the genetic subdivisions identified by the Bayesian tests, as well as a range of other relevant groupings. Permutation tests for two-level AMOVAs were implemented to test whether levels of differentiation were significantly greater than zero. AMOVA results were obtained using GenAlEx6.3 [33].

Assuming that cpDNA does not recombine and can be treated as a single locus, we combined the sizes of all six cpSSR fragments to give the one chloroplast haplotype (chlorotype) per individual. A median-joining network [34] was constructed using NETWORK v4.5.1.6 [35], a program that constructs networks based on size differences among haplotypes.

### Detecting hybridisation

Since STUCTURE (and the other analytical approaches used) is not explicitly designed to assign individuals to admixed classes, we used the Bayesian model implemented in NewHybrids [36] to detect admixed individuals resulting from the interbreeding between distinct species. The original tests and simulations by Anderson & Thompson [36] show that admixture can be detected without the need for diagnostic alleles. Although a high number of informative loci produces much better posterior probabilities (PP) for assigning to hybrid categories, when F_st_ > 0.2 (as is the case in this study; Table 2) a smaller number of loci is sufficient for detecting hybrids [37].

**Table 2:** Diversity values for the taxa investigated

	**Pops**	**N**	**A**	**Ap**	**R**_**63**_	**Mean H**_**o**_	**Mean H**_**e**_	**F**_**st**_**wtn**	**F**_**st**_**btw**	**Mean Q**	**cpSSR**
*T. aspera*	4	84	9.3	18	9	0.554	0.626	0.046	0.265	0.95	1,2
*T. mongaensis*	5	70	6.3	7	6.3	0.38	0.492	0.192	0.318	0.96	4,6
*T. mongaensis* plus *T. oreades* at Monga NP	6	98	6.7	7	6.9	0.415	0.542	0.22	0.303	0.95	4,6
*T. oreades*	8	129	7.3	12	6.8	0.482	0.619	0.238	0.252	0.84	4
*T. oreades* minus *T. oreades* at Monga NP	7	98	6.5	11	6.3	0.472	0.564	0.201	0.276	0.95	4
*T. speciosissima*	19	334	17.2	54	12.7	0.686	0.794	0.112	0.222	0.95	3,4,5,6
*T. speciosissima* south of Shoalhaven only	3	62	8.5	6	7.6	0.648	0.714	0.093	0.243	0.90	6

The rows report species (or groups pf population) specific values. Pops: number of populations; N: number of individuals; A: allelic diversity; A_p_: number of private alleles for the species; R_63_: unbiased allelic diversity; Mean H_e_ and Ho: mean expected and observed heterozygosities; F_st_ wtn: average F_st_ value among populations within a species; F_st_ btw: average F_st_ value relative to other species; Mean Q: mean individual-level assignment value to a species (based on the Bayesian clustering approach implemented in STRUCTURE, at K = 6); cpSSR: identity of chloroplast haplotypes amplified.

Our objective was not to identify and quantify specific admixture categories but rather to ascertain whether differentiated genomes, particularly those of *T. oreades* and *T. mongaensis,* have been mixed where the species overlap. To this end, we conducted a series of preliminary analyses that culminated in a run using all 11 populations from the two species. Jeffreys priors were used with a burn-in of 1x10^5^ sweeps followed by 3.5x10^5^ sweeps. Posterior probability of assignment as pure, F1s, F2s, and backcrosses were initially measured, and proportions of admixture vs. pure were then calculated from these results. An individual was assigned as a pure or hybrid individual when its posterior probability of belonging to that genotypic class exceeded PP = 90% [38,39].

Factorial correspondence analysis (FCA) of diploid genetic data [40] was carried out using GENETIX 4.05 [41] to produce comparative graphical representations of genotypic relationships between all *T. oreades* and *T. mongaensis* individuals sampled.

NewHybrids was also used to analyse the *T. speciosissima* and *T. mongaensis* populations that were geographically closest to the only known morphological intermediate between these two species. This was to serve as a test for the analytical approach and to investigate the possible presence of introgression.

### Current and historical environmental niche modelling

We used the machine-learning automated statistical algorithm Maxent 3.3.3 (Maximum entropy modeling application; [42]) to identify the multivariate correlations between the 19 available ~920 m x 770 m pixel (30 arc-second) WorldClim [43] climate axes (environmental data) and climate conditions present at geographic localities matching the known native distributions of each of the *Telopea* species. We employed this environmental niche modeling (ENM) technique to determine whether present day habitat characteristics correspond to genetic divergence between taxa. Maxent was chosen as it has been shown to outperform other modeling methods when generating predictions of species’ ranges [44].

Occurrence records were compiled from all Australian Herbaria (voucher - high taxonomic integrity) and taxonomically corrected records from the (non-voucher - high geographic integrity) databases: YETI plots [45]; and ATLAS incidental observations [46]. We ran multiple model sets for *Telopea* as a whole and for each *Telopea* species to determine consistent geographic extents between models and consistently dominant climatic factors [47]. Model run 1) only included locality records with accuracies better than 100 m (Table 3). These high precision sites were also of high accuracy (giving ±0.1 climatic pixel accuracy) and those sites that fell within 100 m of the climatic pixel boundary were excluded. This gave a single climatic pixel that best represents the sites sampled. This model represents a minimum estimate of the realised climatic space (climatic conditions in which the species is specifically recorded) of each species but could result in over-fitting of the models [47]. Model 2) included Herbaria records of geographic accuracies better than 1 km, (±1 climatic pixel accuracy or a potential of 9 climatic pixel values per site); and model 3) the above plus non-voucher records better than 1 km accuracy (Table 3). Whilst the climatic data submitted to the model is less precise, substantially more points are submitted providing more robust correlations. Model runs 2) and particularly 3) provide better estimates of the fundamental climatic space (the climatic conditions under which the species is capable of reproducing and persisting) and minimise the effect of false omissions from the training localities which would usually result in models underestimating suitable climatic ranges [47]. The three sets of model runs were compared for between-model consistencies. The larger numbers of localities submitted to the training data better capture the realised climate space and avoid having (climatic) variable numbers approaching the number of sample locations which leads to over-fitting of the models [47].

**Table 3:** Site details for the ENM models

	**Model 1: 100 m Herbarium voucher**	**Model 2: 1 km Herbarium voucher**	**Model 3: 1 km Herbarium and non-voucher**
*T. aspera*	4	14	113
*T. mongaensis*	4	24	150
*T. oreades*	7	37	259
*T. speciosissima*	19	85	647

Numbers of sites analysed for each of the four *Telopea* species per ENM run type.

Model outputs were projected onto to the eastern states of Australia using the same 19 WorldClim variables for 0 ka (pre-industrial) and 21 ka (LGM) time frames. Models were run in the default settings but with 10 replicates under the cross-validation option, and with response curves and jackknife settings enabled. However, this resulted in over-fitted models (as shown geographically by over fragmentation and underestimation of suitable climatic habitat within expected available soil/plant community types) so the models were rerun with hinge features disabled.

The models were run initially with all 19 variables to determine the relative contributions of each environmental variable to the Maxent model. Then, to reduce the effects of overfitting [47], only variables that were not auto-correlated and contributed more than 5% to the Maxent model were included in a subsequent model run.

*Telopea* species are highly constrained by soil type, and non-climatic abiotic factors like this can constrain realised distributions to portions of the fundamental climatic space. If obviously dominant factors of this nature are excluded from the assessment of suitable geographic extents to submit to the training of the models, then this has the effect of submitting more false omissions to the model, producing an underestimation of suitable habitat [47]. To better determine the available realised habitat and estimate the (true) pseudo-absences within the fundamental space, the geographic extents of the training data were clipped to suitable soil types. To make soil type allocation relevant to current and historic time frames, surface geological units were used rather than soil type and were buffered to 1 km to capture likely surface soil types. Soil types for each species were assessed by overlaying occurrence records with soils mapping and resulted in the identification of mostly sedimentary siliciclastic, quartz-rich arenite to rudite or igneous felsic intrusive lithologies.

In order to maintain the integrity of the pseudo-absence reference that is integral to the software and to reduce the effects of over fitting [47], model runs were repeated for the 19 training environmental variables from 0 ka (pre-industrial time) and the geographic extents of training data were constrained to within 200 km of: 1) the genus point locations; 2) the species point locations; 3) the species point locations and to suitable soil type. These three sets of model runs represent increasing removal of false omissions from the training data. Each of these model runs was projected onto the following two geographic extents: a) the eastern Australian states; and b) the eastern Australian states constrained by available suitable soil type. The latter output represented a reduction in the false commissions in the projection.

To determine the extent of overlap between species pairs, the outputs of the last model run (3b) were intersected for each species pair in ArcGIS 9.2 to produce a map layer of the probability of the two species to overlap (Table 4).

**Table 4:** **Modelled current (pre-industrial) and LGM distribution overlap among four *Telopea *species**

**Species**		***T. aspera***		***T. speciosissima***		***T. mongaensis***	
		***p***	**Area (ha) (*p*** **> =0.05)**	***p***	**Area (ha) (*p*** **> =0.05)**	***p***	**Area (ha) (*p*** **> =0.05)**
*T. speciosissima*	LGM	0.142	141				
	Current	0.247	2400				
	% Change	74	1603				
*T. mongaensis*	LGM	0.002	ND	0.361	213299		
	Current	0.004	ND	0.616	498960		
	% Change	100	ND	71	134		
*T. oreades*	LGM	0.033	ND	0.391	6782	0.535	142955
	Current	0.039	ND	0.452	120901	0.202	22041
	% Change	18	ND	16	1683	−62	−85

*p* represents the maximum modeled likelihood of geographic distribution overlap between species pairs within any one pixel. The area (in hectares) represents overlap areas of maximum modeled likelihood of overlap exceeding 5% for both species in the comparison.% Change is the change in species overlap extent from LGM to current. ND is no area detected for *p* > = 0.001 by the 30 arc second pixel models.

## Results

### Defining genetic boundaries among lineages

The seven SSR loci amplified a total of 154 alleles across the 36 populations representing four recognised *Telopea* species. Species-level allelic richness ranged from R_63_ = 6.3 to 17.2, and heterozygosity measures from H_e_ = 0.492 to 0.794 and from H_o_ = 0.380 to 0.686 (Table 2). *Telopea speciosissima* was consistently the most, and *T. mongaensis* consistently the least diverse species. When analysed on their own, the three most southerly populations of *T. speciosissima* still contained high levels of diversity, while shifting the putative *T. oreades* population at Monga NP to *T. mongaensis* made little difference to their respective diversity measures (Table 2).

Average pairwise F_st_ values suggest strongest genetic differentiation within *T. oreades* (particularly when the Monga NP population is included) and weakest within *T. aspera*. *Telopea mongaensis* was the most genetically differentiated from all other taxa (Table 2).

Using the seven nSSR loci, STRUCTURE’s Bayesian clustering produced the most substantial increases in *Ln*P(D) values at K = 5, with the Δ*K* statistic preferentially supporting K = 5 (Δ*K*_5_ = 358.7) as the most robust division. Here we visualise data from the next most robust division (Δ*K*_6_ = 198.5) as it better represents previously-described sub-structure within *T. speciosissima*[10]. Assignment values of Q ≥ 95% were usually obtained at the species level (Table 2). However, two major taxonomic inconsistencies emerged: a) the assignment of the *T. oreades* population at Monga NP to *T. mongaensis*; b) the separation of the southern populations of *T. speciosissima* from the rest of the species.

**Figure 2:**
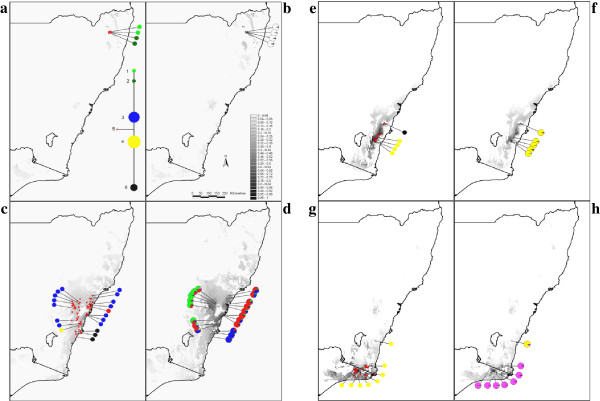
**Modelled bio-climatic distribution of four *Telopea *species (LGM and current) and measured population-level diversity (chloroplast and nuclear).** Maxent modeled bio-climatic distribution for each *Telopea* species for the LGM (21 ka) and current (pre-industrial) time frames (showing only model run 3b being all records projected only onto soils suitable for *Telopea*), showing population-level nSSR and cpSSR (colour-coded haplotypes) data for each of the sampled populations. The nSSR pie charts partition mean individual-level assignment value to a species (based on the Bayesian clustering approach implemented in STRUCTURE, at K = 6). The chloroplast network is shown in (a), and the model probability (likelihood occurrence of suitable habitat) is in a gray scale from 0 (absent) to1 (certain presence) in (b). **a**) *T. aspera*, LGM habitat and cpSSR; **b**) *T. aspera*, current habitat and nSSR; **c**) *T. speciosissima*, LGM habitat and cpSSR; **d**) *T. speciosissima*, current habitat and nSSR; e) *T. mongaensis*, LGM habitat and cpSSR; **f**) *T. mongaensis*, current habitat and nSSR; **g**) *T. oreades*, LGM habitat and cpSSR; **h**) *T. oreades*, current habitat and nSSR.

AMOVA supported significant segregation among *Telopea* species. High levels of genetic variance were partitioned to between-species differentiation (42.7% of variation; P < 0.001), although similar levels of genetic differentiation were partitioned to within-species (43.6% of variation; P < 0.001). A total of six distinct chlorotypes were obtained across the four mainland species and none of the sampled populations amplified more than one chlorotype each (Table 2). A single, shared chlorotype (chlorotype 4) was amplified for all *T. oreades* (Figure 2g)*,* all but one population of *T. mongaensis* (Figure 2e) as well as the Brogers Creek population of *T. speciosissima* (Figure 2c). Multiple chlorotypes were obtained for *T. speciosissima*, with chlorotype 6 being found only among the southern populations as well as the Gunrock Creek population of *T. mongaensis* (Figure 2e).

### Detecting hybridisation

**Figure 3:**
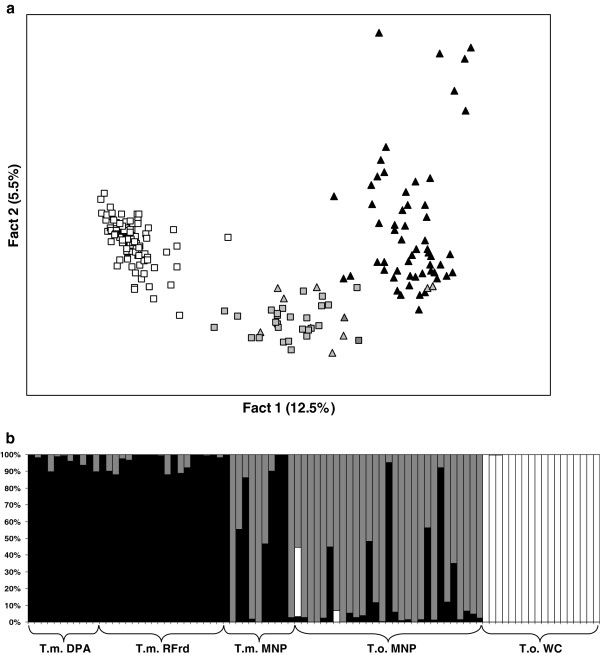
**Testing hybridisation patterns at a distributional overlap zone (*T. oreades *and *T. mongaensis *at Monga NP).a**) Factorial analysis comparing all individuals from all tested populations from *T. oreades* (white squares) and *T. mongaensis* (black triangles). The individuals from the Monga NP site morphologically assigned to either of the two species are represented in grey. **b**) NewHybrids results comparing the individuals (single bars) from the Monga NP site to the geographically closest populations of *T. mongaensis* (Dasyurus PA, River Forest Road) and *T. oreades* (Waratah Creek). *T.m.* DPA: *T. mongaensis* – Dasyurus PA; *T.m.* RFrd: *T. mongaensis* – River Forest Road; *T.m.* MNP: *T. mongaensis* – Monga NP (sympatric); *T.o.* MNP: *T. oreades* – Monga NP (sympatric); *T.o.* WC: *T. oreades* – Waratah Creek. Black represents% assignment to *T. mongaensis* genotype, white represents% assignment to *T. oreades*, grey represents% assignment to hybrid.

Further STRUCTURE analyses were conducted to refine and clarify the interpretations of the assignment among the two southern taxa. Bayesian clustering across 199 individuals from the two southern *Telopea* species, produced substantial increases in *Ln*P(D) values at K = 2 and K = 3, with the Δ*K* statistic preferentially supporting K = 3 (Δ*K*_2_ = 417.4; Δ*K*_3_ = 1009.7). This result identified the southern-most population at Monga NP (including both putative *T. oreades* and *T. mongaensis* individuals) as differentiated from the two species. K = 2 differentiated between the two species but assigned the *T. oreades* population at Monga NP to *T. mongaensis*. The FCA plot provided further insight in the K = 3 result by placing the individuals at the Monga NP site (morphologically identified as either of the parental species or hybrids) as a third intermediate group suggesting either a differentiated lineage or a hybrid population (Figure 3a).

The Bayesian assignment test implemented in NewHybrids correctly identified the only morphologically intermediate individual between *T. speciosissima* and *T. mongaensis* (collected at Brogers Creek) as a hybrid (PP = 92%) when no priors were given, and as an F1 hybrid (PP = 98%) if an F1 prior was used in the analysis. No further sign of admixture was detected between the two species, even among the other individuals at Brogers Creek (average assignment to *T. speciosissima* for the other Brogers Creek individuals; PP = 98%).

NewHybrids analysis of the *T. mongaensis* and *T. oreades* populations identified extensive hybridisation at the Monga NP site (Figure 3b), with 13% of individuals assigned to pure *T. mongaensis* (PP > 90%), and the remainder assigned to a potential hybrid category (PP < 90% of being pure) with 60% having PP > 90% of being either F2 or back-cross to *T. mongaensis*. None of the Monga NP individuals (identified based on morphology to either *T. mongaensis* or *T. oreades*) were assigned to pure *T. oreades* (only four individuals had a PP > 0% of being pure *T. oreades*). At the other sites*,* all individuals (with the exception of three *T. mongaensis* individuals at River Forest Rd, the site closest to the Monga NP site) were assigned with PP > 90% to either pure *T. oreades* or pure *T. mongaensis*.

### Current and historical environmental niche modelling

The average test AUC for the 10 replicate runs indicates excellent model fit for all species tested across all model sets indicating an excellent fit to estimated realised climatic spaces for *Telopea* and for each species tested [47]. For the model sets 1-3b (trained on extents to within 200 km of the species point locations and to suitable soil type, and projected onto only suitable soil types in the eastern Australian states) the average test AUC was: *T. aspera* 0.998 (std dev 0.001); *T. speciosissima* 0.943 (std dev 0.018); *T. mongaensis* 0.990 (std dev 0.006); and *T. oreades* 0.983 (std dev 0.011). However, this does not mean that the models sufficiently estimated the fundamental climatic distributions [47]. The model run for the Herbarium records of *T. aspera* with 100 m accuracy (with lower number of training localities) was the most fragmented, indicating overfitting of the model. Fragmentation of the remaining model sets within the expected distribution was minimal, particularly for the soils runs, indicating good representation of the fundamental climatic space for each species. Interestingly, the distribution of soil types suitable to *Telopea* closely matches the modelled climatic distribution, consequently modelled climatic probabilities and areas did not differ appreciably between model runs, at species or genus levels, with or without soils included. *Telopea aspera* responded most to higher precipitation in the warmest quarter. *Telopea speciosissima* responded to higher mean temperatures for the warmest and wettest quarters and to a higher precipitation in the driest quarter. *Telopea mongaensis* responded to higher temperatures in the wettest quarter and lower temperatures in the driest quarter (low seasonal temperature and precipitation variation). *Telopea oreades* responded to low maximum temperatures (for the warmest month and quarter).

The modelled available bio-climatic habitats for *T. aspera* and *T. speciosissima* expanded from the LGM to present (Figure 2a-d). In contrast, the distribution of habitats suitable for *T. mongaensis* and *T. oreades* declined from the LGM to present (Figure 2e-h). As *Telopea* generally occurs in topographically variable habitats it is probable that there was greater in-situ preservation than the LGM modeling indicates, particularly in microhabitats too fine to be observed in the model pixel size of 30 arc seconds (approximately 850 m).

The LGM bio-climatic models for the various lineages support appreciable overlap only for *T. speciosissima* and *T. mongaensis*, and *T. mongaensis* and *T. oreades*. Due to the modelled current expansion of *T. aspera* and *T. speciosissima,* overlap of distributions increased between all species pairs excluding between *T. mongaensis* and *T. oreades* (Table 4). Although some low-level potential overlaps are modelled between these two species, a 160 km wide distributional gap currently exists. Similarly, despite a large potential habitat overlap between *T. speciosissima* and *T. oreades* they are significantly separated geographically. Similarly *T. aspera* is currently too geographically disjunct (400 km) from all species for any interaction to occur.

## Discussion

### Concordance between geographic range and the distribution of genes and suitable habitat

The species boundaries described for *Telopea*[7] follow a pattern of latitudinal differentiation along the Great Dividing Range (GDR). The GDR, running north–south along the length of eastern Australia, is a dominant topographic feature. Despite having relatively low elevation, it is sufficiently close to the coast to provide precipitation gradients. These gradients support a range of species and communities that differ considerably from those found further west across the prevalently dry continent. The GDR supported refugial habitats during the climatic oscillations of the Quaternary, as revealed by regional species turnover [48,49] and deep between-population genetic divergences [50,51]. Based on palaeoecological evidence [12,13], phylogeographic divergences are interpreted as the genetic signatures of distributional contractions caused by climate-induced loss of suitable habitat. Habitat contractions were followed by re-expansions at the return of more favourable conditions [52-54]. These recurring Quaternary patterns have the potential to isolate populations and initiate allopatric speciation, even in the absence of strong intrinsic reproductive barriers [55,56].

The nSSR data provided fine-scale resolution and broad support for latitudinal species-level genetic divergences (Figure 2). The highest diversity was measured in the most widespread species (*T. speciosissima*) and, as expected in an allopatric speciation model [57], the more diverse species also showed signs of significant structure particularly in relation to latitudinal landscape barriers and elevation. Current environment niche models are also in agreement with species being differentiated along the latitudinal gradient, suggesting relatively small bio-climatic overlaps between species (Table 4). Genetic and bio-climatic boundaries correspond to breaks in the GDR. *T. aspera* and *T. speciosissima* are separated by the Hunter River Corridor (an important floristic boundary; [58,59]). With the exception of the *T. oreades* population at Monga NP, *T. mongaensis* and *T. oreades* are separated by an elevation decline of the GDR (previously reported as affecting genetic structure in other plants; [60]) and the fragmented nature of suitable edaphic conditions.

Chloroplast SSRs could not fully resolve species boundaries (only six chlorotypes were discovered across the entire distribution of the four continental species), however the geographic distribution of the chlorotypes confirmed a latitudinal partitioning of genetic variation, even confirming within-species latitudinal divergences in *T. speciosissima* (Figure 2). Thus, genetic and bio-climatic analyses largely corroborate an allopatric speciation model where the latitudinal gradient is a major factor influencing lineage divergence. Nevertheless, present-day distributions include potential contact zones between species, and localised chlorotype-sharing suggests that between-species admixture is possible. If confirmed, hybridisation events could provide supporting evidence either for secondary contact between once-disjunct taxa or for parapatric differentiation along the borders separating sister taxa. Consequently, we explored in greater detail the possible occurrence of hybridisation at known contact zones.

### Supporting evidence for hybridisation at contact zones

Artificial hybrids among *Telopea* species are common in the horticultural trade and all species are known to be potentially inter-fertile in cultivation [61]. A single and isolated individual that is morphologically intermediate between *T. speciosissima* and *T. mongaensis* was confirmed as a hybrid (potentially an F1) by the nSSR analyses. No *T. mongaensis* individuals were found in the vicinity, and the closest *Telopea* population was *T. speciosissima* at Brogers Creek. Interestingly, the Brogers Creek population shared chlorotype 4 with *T. mongaensis* despite bearing no morphological or nuclear signature of admixture. Furthermore, while the nSSR data preferentially assigned this same population to the southern *T. speciosissima* group, the geographically closest *T. mongaensis* population shared chlorotype 6 with the southern *T. speciosissima*, while being unequivocally assigned to *T. mongaensis* by nSSRs (Figure 2). These patterns suggest temporal variations in species overlap in response to changing climatic conditions, leaving genetic signatures similar to those observed in Tasmanian eucalypts [62,63].

*Telopea oreades* and *T. mongaensis* are sister taxa and the only qualitative morphological character that differentiates them is the absence of foliar sclereids in *T. mongaensis.* They also differ in average leaf dimensions, bract width and prominence of leaf venation but variation in these characters overlaps between the species [8]. Consequently, morphological intermediates are difficult to characterise. These two species are generally geographically disjunct, but variation in leaf morphology, including the presence of foliar sclereids in large-leaved plants, suggested that *T. oreades* and *T. mongaensis* may occur in sympatry at Monga NP (the southern distributional end of *T. mongaensis*). While both species share chlorotype 4 (Figure 2), which could be interpreted as a result of incomplete lineage sorting in view of limited chloroplast variation, the nSSR-based Bayesian analyses implemented in NewHybrids presented an unambiguous admixture scenario at Monga NP (Figure 3) suggesting repeated backcrossing to *T. mongaensis* but no evidence of the persistence of pure *T. oreades*. Interestingly, when Crisp & Weston [8] excluded sclereids from their morphometric analysis, individuals sampled from Monga NP spanned the full range of morphological variation between pure *T. mongaensis* and pure *T. oreades*. Their results were consistent with our genetic evidence but when viewed through the lens of a categorical distinction between plants possessing or lacking sclereids, morphological variation at Monga NP was interpreted as circumscribing two sympatric, albeit sporadically hybridising species rather than a single, morphologically variable population [8].

Rather than a scenario of parapatric speciation, where hybrid zones continuously occur along adaptive clines at the distributional extremes of species [3], our findings are more supportive of a scenario similar to the one proposed for *T. speciosissima* and *T. mongaensis*. Temporal climatic fluctuations caused distributional changes that resulted in repeated cycles of secondary contact. As suitable habitat contracted, the *T. oreades* population at Monga NP remained isolated and was integrated by *T. mongaensis*. A history of allopatric speciation is unlikely to result in rapid and strong postzygotic reproductive isolation and consequently, complex hybridisation events can be expected in secondary contact zones [1]. However, further evidence for temporal changes in connectivity was needed in order to exclude a scenario of parapatric speciation.

### Bio-climatic evidence for temporal changes in connectivity

A previous study on *T. speciosissima* suggested that the genetic differentiation measured among population groups representing coastal, upland and southern distributions was the consequence of cyclical distributional adjustments during the Quaternary [10]. An exploration of temporal changes in the availability of suitable habitat supports contractions into refugia during the LGM (Figure 2). In fact, Hesse *et al*. [64] suggested that local environmental conditions were so extreme during the LGM that upland areas were largely devoid of woody vegetation.

Unexpectedly, whilst the temporal distributional variation that provides evidence for intra-specific allopatric differentiation in *T. speciosissima* is characterised by habitat contractions during the LGM (also evident for its northern sister species, *T. aspera*), the circumstances are reversed for the two southern taxa (Figure 2). Current interglacial bio-climatic conditions confirm the existence of an inter-specific distributional gap, while modelled LGM ENMs provide support for expansions of suitable habitat. The LGM models support the scenario of a *T. oreades* x *T. mongaensis* hybrid population at Monga NP, currently disjunct from other *T. oreades* populations and likely to have originated from historical habitat expansions resulting in a now-vanished overlap zone.

Overall, the bio-climatic models show that cyclical contractions/expansions of suitable habitat for all *Telopea* are likely to have generated temporally variable inter-specific distribution gaps consistent with allopatric differentiation. Contraction to small population size and the selection for even small adaptive differences would have enhanced the chance of faster stochastic allopatric differentiation [6]. Conversely, there is no evidence for the persistence of the continuous contact zones essential to a parapatric differentiation model [1].

## Conclusions

Although the traditional separation of speciation processes into allopatric and parapatric categories might not capture the full complexity of spatial relationships among taxa [65], our study shows that the evidence for temporal exclusion of gene flow can be found even outside obvious geographical contexts (such as continental drift and island radiations for example). In *Telopea*, between-species differentiation has been regulated mostly by temporal changes in bio-climatic conditions that have repeatedly disrupted the continuum in distribution of the various evolving lineages.

We also show that it is possible to make significant progress towards excluding parapatric speciation as a contributing evolutionary process in a selected study system. *Telopea* includes allopatric taxa that are at different stages of speciation involving some ecological adaptation but no complete reproductive isolation. Niche differentiation does not imply the establishment of fixed reproductive barriers [3], and the combination of species-specific bio-climatic envelopes and hybrid zones suggests that selective filters shaped local adaptive distinctiveness as a secondary process rather than being the driver of speciation via the establishment of selection-based reproductive barriers.

Current studies involving fine-scale coalescent analyses and transcriptome-based investigations of differential adaptive potential among *Telopea* populations will bring further insights on speciation mechanisms operating in *Telopea*. Comparing genome-level patterns of diversity between populations can facilitate the identification of genomic regions that do not conform to the expectation of neutral demographic models. However, strong divergent selection at a small number of loci would not be expected in an allopatric speciation scenario. Instead, analogous coalescent patterns across different loci could identify the signature of past periods of allopatry. For instance, targeting multiple low recombination regions has been shown to be particularly useful in describing the contribution of allopatric processes to sympatric divergences [66].

## Competing Interests

The authors have no financial or other competing interest to declare.

## Authors' contributions

MR, MLM and PHW conceived and designed the study and collected samples; KAGT and CBA contributed laboratory and modeling data, MLM collected samples and contributed cpDNA data and analyses; MR analysed the nDNA molecular data and prepared the manuscript with all authors editing and approving the final manuscript.
